# The effects of substance P and acetylcholine on human tenocyte proliferation converge mechanistically via TGF-β1

**DOI:** 10.1371/journal.pone.0174101

**Published:** 2017-03-16

**Authors:** Gloria Fong, Ludvig J. Backman, Håkan Alfredson, Alex Scott, Patrik Danielson

**Affiliations:** 1 Dept. of Integrative Medical Biology, Anatomy, Umeå University, Umeå, Sweden; 2 Centre for Hip Health and Mobility, Vancouver Coastal Health Research Institute, Vancouver, BC, Canada; 3 Dept. of Community Medicine and Rehabilitation, Sports Medicine, Umeå University, Umeå, Sweden; 4 Dept. of Clinical Sciences, Ophthalmology, Umeå University, Umeå, Sweden; Weizmann Institute of Science, ISRAEL

## Abstract

Previous *in vitro* studies on human tendon cells (tenocytes) have demonstrated that the exogenous administration of substance P (SP) and acetylcholine (ACh) independently result in tenocyte proliferation, which is a prominent feature of tendinosis. Interestingly, the possible link between SP and ACh has not yet been explored in human tenocytes. Recent studies in other cell types demonstrate that both SP and ACh independently upregulate TGF-β1 expression via their respective receptors, the neurokinin 1 receptor (NK-1R) and muscarinic ACh receptors (mAChRs). Furthermore, TGF-β1 has been shown to downregulate NK-1R expression in human keratocytes. The aim of this study was to examine if TGF-β1 is the intermediary player involved in mediating the proliferative pathway shared by SP and ACh in human tenocytes. The results showed that exogenous administration of SP and ACh both caused significant upregulation of TGF-β1 at the mRNA and protein levels. Exposing cells to TGF-β1 resulted in increased cell viability of tenocytes, which was blocked in the presence of the TGFβRI/II kinase inhibitor. In addition, the proliferative effects of SP and ACh on tenocytes were reduced by the TGFβRI/II kinase inhibitor; this supports the hypothesis that the proliferative effects of these signal substances are mediated via the TGF-β axis. Furthermore, exogenous TGF-β1 downregulated NK-1R and mAChRs expression at both the mRNA and protein levels, and these effects were negated by simultaneous exposure to the TGFβRI/II kinase inhibitor, suggesting a negative feedback loop. In conclusion, the results indicate that TGF-β1 is the intermediary player through which the proliferative actions of both SP and ACh converge mechanistically.

## Introduction

Tendinosis is a chronic condition involving histological changes in the tendon structure [1]. It has been shown that substance P (SP) and acetylcholine (ACh), signal substances traditionally thought to be confined to the neuronal system, may contribute to the pathophysiology of tendinosis as the levels of these substances are elevated in tendinosis tendons as compared to in normal tendons [2–5]. Elevated levels of SP precede tendinosis [6], suggesting that SP not only maintains tendinosis, but is also involved in the development of the condition. In previous studies on human tenocytes *in vitro*, exogenous administration of both SP and ACh independently caused increased cell proliferation and metabolism [7, 8]. The similar proliferative actions of SP and ACh are unlikely to be coincidental, but rather the result of the two signal substances sharing the same mechanistic pathways downstream. However, this potential convergence of pathway has not been studied in human tenocytes.

It has been shown in other studies that both SP and ACh independently induce transforming growth factor beta-1 (TGF-β1) expression. In human lung epithelial cells, the ACh analogue carbachol induces TGF-β1 expression in a dose-dependent manner via activation of mAChR, while this effect was blocked by a muscarinic receptor blocker, atropine [9]. Another study has shown that stimulation with exogenous SP induces TGF-β1 expression in lung epithelial cell lines in a dose-dependent manner [10]. Moreover, TGF-β1 has been shown to downregulate the expression of NK-1 R, the preferred receptor for SP, in corneal stromal cells, keratocytes [11]. The aforementioned studies suggest that the effects of TGF-β1 may be the result of the upstream influence by SP and ACh.

Based on the recent studies as highlighted above, it can be conceptualized that TGF-β1 is a potential candidate involved in the effects of SP and ACh, possibly converging mechanistically in tenocytes. TGF-β1 is known to be essential for tendon growth and adaptation via its role in promoting tenocyte proliferation and collagen I synthesis [12]. As well, TGF-β1 is responsible for upregulating tenomodulin, a type II transmembrane glycoprotein that is essential for tenocyte proliferation and maturation [13]. As TGF-β plays a vital role in mediating tenocyte proliferation and maturation, dysregulation of the TGF-β axis has been implicated in tendinosis [14].

TGF-β is a cytokine with three isoforms in mammals, TGF-β1, TGF-β2 and TGF-β3. These three isoforms are structurally similar but are encoded by different genes and exert their effects by binding to the TGF-β receptors which can be subdivided into three classes, type I, type II and type III receptors (TGF-βR I, TGF-βR II and TGF-βR III respectively) [14]. To initiate signalling, the TGF-β ligand first binds to TGF-βR II, which then creates a receptor complex with TGF-βR I. This formed receptor complex results in the phosphorylation of TGF-βRI by TGF-βRII which leads to its activation [14]. Upon activation, the receptor complex initiates an intracellular cascade involving the TGF-β specific phosphorylation of SMAD and formation of SMAD complexes [14]. There have been several studies conducted that show TGF-β1 to induce tenocyte proliferation in the *in vitro* setting [15, 16].

In view of the above, we hypothesize that the downstream effects of SP and ACh converge mechanistically and that TGF-β1 is the intermediary player associated with the proliferative effects of SP and ACh. For this hypothesis to be tested, the following questions were studied: (1) If SP and ACh cause upregulation of TGF-β1 in human tenocytes, (2) if TGF-β1 exerts proliferative properties in human tenocytes, (3) if the proliferative effects of SP and ACh are reduced by TGF-βRI/II kinase inhibitor, and (4) if TGF-β1 downregulates NK-1 R and mAChR, suggesting the presence of a negative feedback mechanism.

## Materials and methods

### Primary culture of human tendon cells

Achilles tendon biopsies, derived from the mid-portion of healthy human donors, were harvested. Healthy donors were defined as individuals having no history of Achilles tendon pain, and clinical and ultrasound + Colour Doppler examination showing normal findings. The biopsies were minced and cultured as previously described to establish primary tendon cell cultures [8]. Briefly, the biopsies were enzymatically digested with 2 mg/ml collagenase (Sigma, Clostridopeptidase A, C-0130) for 120 minutes and cultured in D-MEM (Invitrogen, 11960) supplemented with 10% fetal bovine serum (FBS) (Invitrogen, code: 16000), 1% penicillin/streptomycin (Invitrogen, code: 25030) and 0.2% L-glutamine (Invitrogen, code: 25030) at 37°C in 5% CO_2_ as previously described [8]. A total number of six Achilles tendon samples were used in the study.

### Experimental conditions

The primary tendon cells used in this study were from passages 3 to 6 to ensure similar phenotype as characterized in our previous studies [7, 8]. All experiments were conducted under overnight serum-starved condition in 1% FBS containing DMEM media similar to prior studies [8, 17].

### Reagents

TGF-β1 (R&D Systems, code: 240-B-002), reconstituted in 4mM hydrochloric acid and 0.1% bovine serum albumin (BSA, Sigma, code: A9647) was used at a concentration of 1 ng/ml based on modification from Le Roux et al’s study involving human keratocytes [11]. The TGFβRI/II kinase inhibitor LY2109761 (Santa Cruz, code: sc-396262) was used at a concentration of 2 μmol/l based on Xu et al’s study [18]. LY2109761, prepared in dimethyl sulphoxide (DMSO), was used to block the effects of TGF-β1, and was added 20 min prior to TGF-β1 incubation. In addition, SP (Calbiochem, code: 05-23-0600) at 10^−7^ M and NK-1 R receptor blocker (L-733.060) (Tocris, code: 1145) at 10^−6^ M were used in concentrations as previously used in Backman et al’s study involving tenocytes [8]. SP at 10^−7^ M has also been used in studies involving human colonocytes [19] and human lung epithelial cell lines [10]. The concentrations of the mAChR blocker atropine at 10^−5^ M (Sigma, code: A0132) and ACh at 10^−6^ M (Sigma, code: A661) were based on our previous study involving human tenocytes [7].

### Human TGF-β1 immunoassay

The TGF-β1 immunoassay (ELISA) was performed according to the manufacturer’s protocol (R&D, code: DB100B). Cells were frozen at -80°C at the designated time-points until the ELISAs were performed. The cells were subsequently lysed in a lysis buffer (RIPA) containing 150 mM sodium chloride, 1% Triton, 0.5% sodium deoxycholate, 0.1% sodium dodecyl sulphate (SDS), 50 mM TRIS, pH 8.0 and supplemented with a protease inhibitor cocktail at a dilution of 1:200 (Sigma, code: P1860).

Briefly, extracted protein was activated by 1 N hydrochloric acid, followed by neutralization with 1.2 N sodium hydroxide/0.5 M HEPES. Following the assay procedure, the optical density of each reaction was measured at 450 nm using a microplate reader (Synergy HT Multi-Mode Reader), and corrected against absorption at 570 nm.

### Western blot

Tendon cells were lysed in RIPA lysis buffer. Protein concentration was quantified by the use of Protein Assay Dye Reagent (Bio-Rad, code: 500–0006) with bovine serum albumin (BSA, Sigma, code: A9647) as a standard. Total protein from each sample was diluted in Laemmli Sample Buffer (Bio-Rad, code: 161–0737), supplemented with beta-mercaptoethanol, and heat denaturated at 95°C for 5 min before loading and separation on a SDS-polyacrylamide gel and subsequent transfer to a polyvinylidene difluoride membrane (PVDF; Sancta Cruz, code: sc-3723). Ponceau S staining (0.1% Ponceau red, 1% acetic acid diluted in Milli-Q water) was used to confirm successful transfer of proteins onto the membrane prior to washing and subsequent blocking in 5% BSA at room temperature for 1 h. The primary antibodies toward NK-1R and TGF-β1 were incubated overnight at 4°C at a dilution of 1:4000 (Sigma-Aldrich, code: s8305) and 1:200 (Abcam, code: ab9758) respectively. The dilution of antibody towards NK-1 R was used previously in Backman et al’s study involving human tenocytes *in vitro* [8]. The concentration of antibody against TGF-β1 was based on the manufacturer’s recommendation. The membranes were subsequently washed and incubated with horseradish peroxidase conjugated secondary antibody at a dilution of 1:2000 (Cell Signaling, code: 7074) for 1 h at room temperature. Band detection was performed using a chemiluminescent HRP substrate for 5 min prior to visualization using the Odyssey Fc Dual-Mode Imaging System (LI-COR Biosciences). To ensure equal protein loading, the primary and secondary antibodies on the membrane were removed with stripping buffer (Thermo Scientific, code: 21059) and re-probed for β-actin (Cell Signaling, code: 4967). Densitometry was performed using ImageJ analysis software (NIH).

### RNA isolation, reverse transcription, and qPCR

RNA isolation, reverse transcription and RT-PCR of Achilles tendon cell cultures were performed as previously described [8]. RNA isolation was performed using the Qiagen RNeasy kit (Qiagen, code: 74106) and extracted RNA was reversed transcribed into cDNA using a High Capacity cDNA Reverse Transcription kit (Applied Biosystems (ABI), code: 4368813). A total sample volume of 14.2 μl containing RNA and RNase free water, was added to 2μl 10 X RT buffer, 0.8 μl 25x dNTP, 2μl 10 x RT random primers and 1 μl 20x multiscribe RTase to obtain a final volume of 20 μl. RNA conversion to cDNA was performed according to the manufacturer’s recommendations: 10 min at 25°C followed by 120 min at 37°C and 5 min at 85°C before idling for 60 min at 4°C in a thermal cycler (MJ Research PTC-200 Thermal Cycler). Quantitative PCR was performed using the TaqMan Fast Universal PCR Master Mix (Applied Biosystems, code: 4352042) by splitting the starting volume of 25μl into two 10μl volumes in each level for technical duplicate. The levels of TGF- β1 (ABI, code: Hs00998133), TAC1 (ABI, code: Hs00243225), TACR1 (ABI, code: Hs00185530), CHRM1 (ABI, code: Hs00912795), CHMR2 (ABI, code: Hs00265208), CHRM3 (ABI, code: Hs00265216), CHMR4 (ABI, code: Hs00265219), and CHRM5 (ABI, code: Hs00255278) were determined relative to β-actin (ABI, code: 4333762F). The amplification conditions include initial denaturation at 95°C for 20 sec, followed by 40 cycles of denaturation at 95°C for 1 sec and annealing/extension at 60°C for 20 sec using the ViiA™ 7 Real-Time PCR System (ABI).

### MTS proliferation assay

Cells were seeded at a density of 5000 cells per well in a 96-well plate. The cells were grown overnight before serum starvation for 24h prior to incubation with TGF-β1 at 1 ng/ml with or without LY2109761 at 2 μmol/l for 48h. The MTS assay (CellTiter 96® AQ_ueous_ One Solution Cell Proliferation Assay System, Promega, code: G3582) was used according to the manufacturer’s recommendations. Briefly, media was removed from all wells and replaced with media containing 20 μl of the MTS reagent in 100 μl of the starvation media per well. After 1h of incubation, the absorbance was measured at 490 nm with a microplate reader (Synergy HT Multi-Mode Reader). Each treatment group consisted of eight wells.

We have in previous studies, on human derived tenocytes, shown that increased MTS correlates to increased crystal violet and bromodeoxyuridine (BrdU) [7, 8].

### Crystal violet cell viability assay

Crystal violet stains total protein and DNA, which would indirectly correspond to the number of cells, and was used to evaluate proliferative effects of substances. Cells were seeded at a density of 1.5x10^5^ cells per well in 6-well plates. The cells were grown overnight before serum starvation for 24h prior to incubation with TGF-β1 at 1ng/ml. Following 24h and 48h, the TGF-β1 and control groups were washed in PBS to remove non-adherent cells prior to fixation in 1% glutaraldehyde for 30 min. After additional washes in PBS, the adherent cells were stained with 1ml 0.1% crystal violet (Sigma, code: C3886), washed in water and subsequent air-dried. The cells were permeabilized in 30% methanol and 10% acetic acid. 150 μl of the solution from each well was transferred to a 96-well plate and the absorbance was read at 590 nm. Experiments were performed in triplicates.

We have in previous studies, on human derived tenocytes, shown that increased crystal violet correlates to increased MTS and BrdU [7, 8].

### Bromodeoxyuridine (BrdU) incorporation ELISA

ACh and SP effects on cell proliferation were performed by measuring BrdU incorporation in newly synthesized cellular DNA according to the manufacturer’s instructions (Roche, code: 11 647 229 001). 1 x 10^4^ tenocytes were seeded into each well of a 96-well plate. Cells were incubated overnight and afterwards treated with ACh (10-^6^M) and SP (10-^7^M). In addition, cells were pre-treated with TGF-βI/II kinase inhibitor (2 μmol/l) as described above. 6 hours following treatment, cells were labelled with BrDU and incubated for 2h at 37°C in 5% CO_2_. The cells were subsequently fixed and anti-BrdU-POD was added for 1h to bind the BrdU incorporated DNA. Finally, a substrate solution was added for 20min before detecting the absorbance at 370 nm (reference wavelength: 492nm) with a micro-plate reader (Synergy HT Multi-Mode Reader).

### Ethics statement

The Regional Ethical Review Board in Umeå approved the study. The study was performed according to the principles of the Declaration of Helsinki. Written informed consent was received from all participants.

### Statistics

Data were analyzed with the GraphPad PRISM 6 software and expressed as mean +/- SD. For comparing more than two treatment groups, one-way ANOVA with Bonferoni post-hoc test was used. When only comparing two groups, student´s t-test was performed. Significance was predetermined at p<0.05.

## Results

### Effects of SP and ACh on TGF-β1 expression

**Fig 1:**
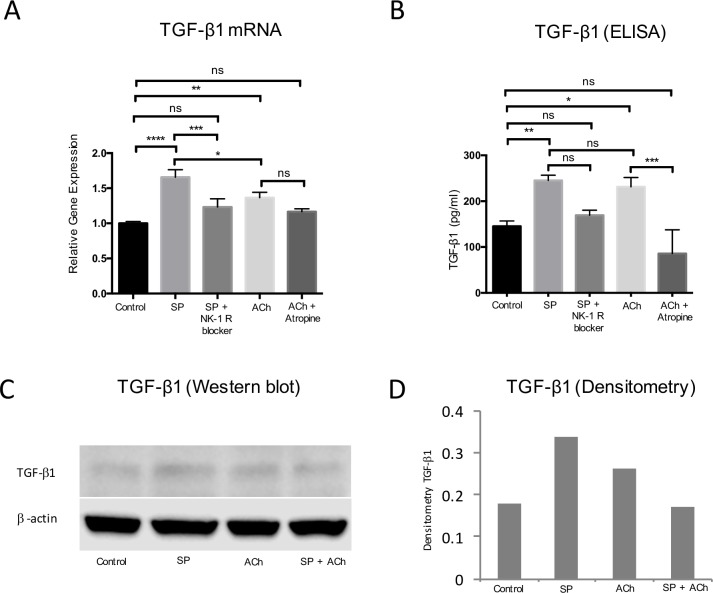
TGF-β1 mRNA and protein expression following SP and ACh exposure in cultured human tenocytes. (A) SP (10^−7^ M) and ACh (10^−6^ M) both independently increased TGF-β1 mRNA expression following 24h of exposure, and the effect of SP was significantly reduced when the NK-1R blocker (10^−6^ M) was included, whereas the ACh effect was not significantly reduced by the mAChR receptor blocker atropine (10^−5^ M). (B) Similar results were observed at the protein level for TGF-β1 as demonstrated by the ELISA assay after 48h. (C) In addition, western blot confirmed that both SP and ACh cause an increased expression of TGF-β1 after 48h, although no additive effect was seen by exposure of both SP and ACh simultaneously. (D) Densitometry of western blot results (Fig 1C) is shown. Values are means ± SD. n.s. (not significant). *p<0.05, **p<0.01, ***p<0.001.

The *in vitro* effects of SP and ACh on TGF-β1 expression were examined at the mRNA level using qPCR and at the protein level using ELISA and western blot analysis. Exogenous administration of SP and ACh for 24h both led to significant increase in TGF-β1 mRNA expression in the cultured tenocytes. This effect of SP was blocked by inhibition of the receptor for SP, the NK-1R (Fig 1A). The expression of TGF-β1 mRNA was significantly higher after SP treatment as compared to after ACh treatment. Similar results were also seen on the level of protein using ELISA at 48h, i.e. that both SP and ACh independently increased the expression of TGF-β1; these effects were blocked by the specific inhibitors of NK-1R and mAChRs (atropine), respectively (Fig 1B). Western blot furthermore confirmed that both SP and ACh induced increased TGF-β1 protein expression after 48h on the protein level. However, there seemed to be no additive increase by exposure of both SP and ACh simultaneously (Fig 1C and 1D).

### Effects of TGF-β1 on tenocyte metabolism and proliferation

**Fig 2:**
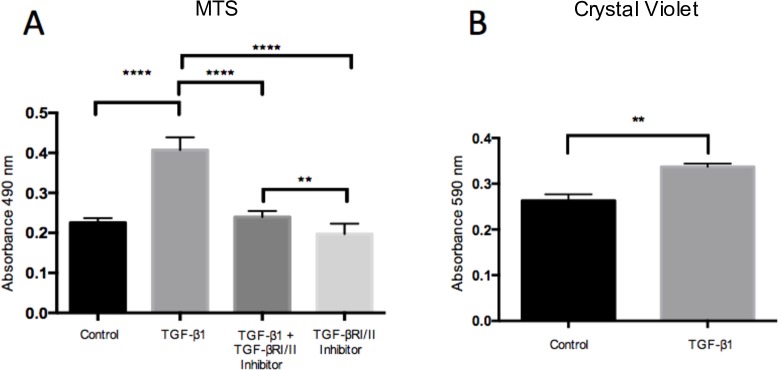
The effects of TGF-β1 exposure on cultured tenocytes as demonstrated by the MTS. (A) and crystal violet (B) assays at 48h. (A) TGF-β1 (1 ng/ml) stimulation resulted in a 1.8-fold significant increase in cell metabolism as compared to control. The TGFBRI/II kinase inhibitor (2 μmol/l) reduced this effect significantly. (B) TGF-β1 resulted in a significant increase in the total number of cells as compared to control, using the crystal violet assay, at 48h. Values are means ± SD. **p<0.01. ****p<0.0001

The effects of TGF-β1 on cultured tenocyte metabolism and proliferation were determined by the MTS assay and crystal violet staining, respectively. Following 48h of TGF-β1 exposure, there was a 1.8-fold significant increase in cell metabolism, as shown with the MTS assay, as compared to control. At 24h, there was no statistical significance between the treatment groups relative to the control (data not shown). The effect of increased cell metabolism was reversed by inhibition of the TGFBRI/II kinase (Fig 2A). The proliferative effect, as measured with crystal violet method, was significantly increased after cells were exposed to TGF-β1 after 48 hours (Fig 2B).

### Effects of SP and ACh on tenocyte proliferation in the presence of TGF-βRI/II inhibition

**Fig 3:**
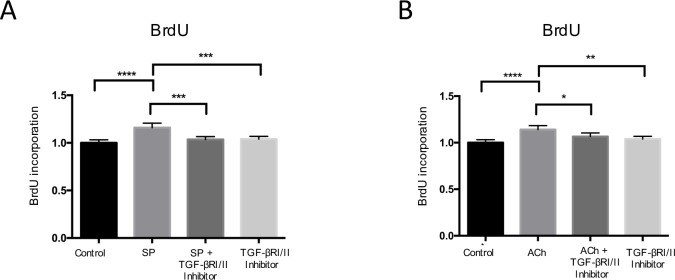
The effects of SP and ACh on tenocyte proliferation in the presence of TGF-βRI/II inhibition as measured by DNA incorporation using the BrdU assay. (A) SP (10^−7^ M) resulted in significant increase in cell proliferation as compared to the control. Relative to SP treatment alone, there was a significant decrease in cell proliferation after pre-treatment with TGF-βRI/II kinase inhibitor (2 μmol/l). (B) ACh (10^−6^ M) led to significant increase in cell proliferation as compared to the control. The significant increase in proliferation seen after incubation with ACh (10^−6^ M) was effectively blocked after pre-treatment with TGF-βRI/II kinase inhibitor. Values are means ± SD. *p<0.05, **p<0.01, ***p<0.001, ****p<0.0001

The effects of SP and ACh on tenocyte proliferation *in-vitro* in the presence of TGF-βRI/II inhibition were measured by the BrdU assay. Pre-treatment with TGF-βRI/II kinase inhibitor significantly reduced the proliferative effects of both SP and ACh (Fig 3A and 3B).

### Effects of TGF-β1 on mAChR and NK-1R expression

**Fig 4:**
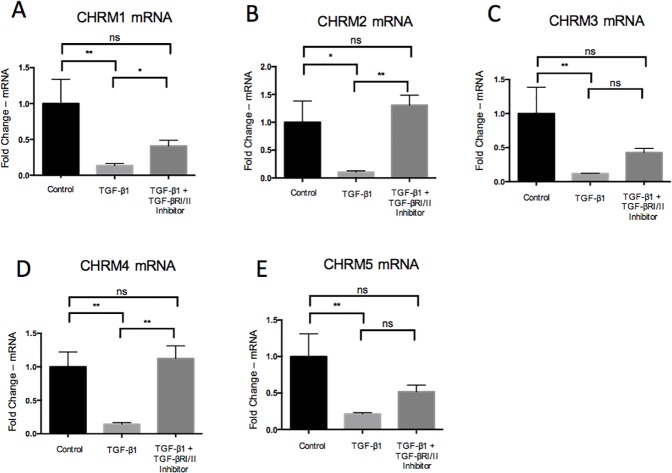
The effect of TGF-β1 on mAChR mRNA expression, for all receptor subtypes (CHRM1 –CHRM5), as measured by qPCR. All mAChRs (CHRM1 –CHRM5) were downregulated following 48h of TGF-β1 (1ng/ml) exposure. The TGFBRI/II kinase inhibitor (2μmol/l) reversed the TGF-β1 downregulation of the muscarinic receptors. Values are means ± SD. n.s. (not significant). *p<0.05 **p<0.01.

**Fig 5:**
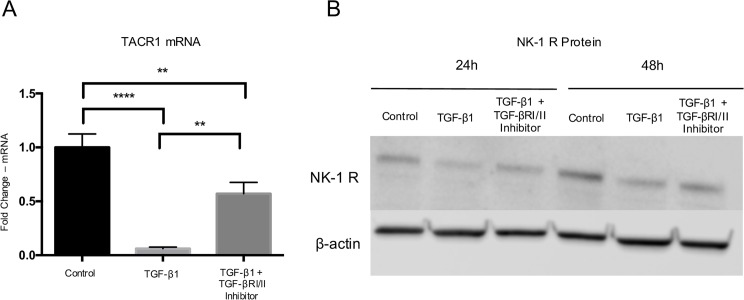
The effect of TGF-β1 on TACR1 (NK-1R) mRNA expression, as measured by qPCR, and on the level of NK-1R protein, as shown by western blot. (A) The TACR1 mRNA expression was significantly decreased with TGF-β1 (1ng/ml) at 48h post exposure. In the presence of the TGFBRI/II kinase inhibitor (2μmol/l), the downregulation was reduced (B) As shown by western blot analysis, the expression of NK-1R was reduced in the TGF-β1 treated group as compared to the control following 24h and 48h. This reduction was hampered in the presence of the TGF-βRI/II kinase inhibitor. Values are means ± SD. p**<0.01, p****<0.0001.

The effects of TGF-β1 on mAChR and NK-1R expression were measured at the mRNA and protein levels using qPCR and western blot respectively. TGF-β1 exposure for 48h resulted in a significant downregulation of all muscarinic receptors (mAChR 1–5) on the mRNA level. The effect was reversed by incubation of TGF-β1 together with the TGFBRI/II kinase inhibitor. Co-administration of TGF-β1 with the inhibitor led to significant increase in expression of CHRM1, CHRM2 and CHRM4 as compared to TGF-β1 alone (Fig 4A–4E). Furthermore, TGF-β1 resulted in significant downregulation of TACR1 (i.e. NK-1R gene) expression 48h post-treatment, while the effect was reversed by TGFBRI/II inhibition (Fig 5A). Similar results were seen on the protein level, i.e. TGF-β1 treatment resulted in a downregulation of NK-1R expression after 24h and 48h, and the effect was reversed when the TGFBRI/II kinase inhibitor was included (Fig 5B).

## Discussion

We hypothesized that the reason that SP and ACh have similar mechanistic effects with regards to increasing proliferation in human tenocytes is the result of the two signal substances converging via a common TGF-β pathway. This is the first study to demonstrate that exogenous administration of SP and ACh both result in upregulation of TGF-β1 in human tenocytes *in vitro*. In addition, exogenous TGF-β1 administration, via its preferred receptor, resulted in increased cell metabolism and proliferation. The proliferative effect of SP and ACh was significantly reduced in the presence of TGF-βRI/II kinase inhibitor. Finally, exogenous TGF-β1 downregulated both NK-1R and mAChRs expression and these effects were negated by simultaneous exposure of the TGFβRI/II kinase inhibitor. The results of the study support the hypothesis that both SP and ACh converge mechanistically and that TGF-β1 is the intermediary player associated with the proliferative effects of SP and ACh.

### SP and ACh independently upregulate TGF-β1 expression, and the proliferative effects of SP and ACh are mediated via the TGF-β axis

SP and ACh resulted in significant upregulation of TGF-β1 at the mRNA and protein levels in tenocytes. Furthermore, inhibition of the NK-1R and mAChRs, resulted in the downregulation of TGF-β1 expression. This finding is consistent with previous reports in other cell types that both ACh and SP can independently induce TGF-β1 expression via their respective receptors [9, 10].

In previous studies, we have shown that both SP and ACh independently stimulate tenocyte proliferation mediated via ERK1/2 [7, 8]. Interestingly, TGF-β1 has also been shown to induce is ERK 1/2 [20]. The fact that SP, ACh and TGF-β1 share at least one downstream mechanism, i.e. ERK1/2 activation, may further support the notion that the proliferative effect of SP and ACh can be mediated via the TGF-β axis. To substantiate this, further studies will be required. In the current study, we demonstrated that the TGF-β axis is intimately involved in the proliferation of tenocytes, as the proliferative effects of both SP and ACh were significantly reduced in the presence of TGF-βRI/II kinase inhibition. Our findings suggest that the activation of NK-1R and mAChRs in tenocytes are only the initial steps to induce tenocyte proliferation, and that the TGF-β axis is essential downstream.

To verify that ACh, SP and TGF-β1 induce tenocyte proliferation, three different assays, the MTS, crystal violet and BrdU assays were used to capture the proliferative process at various time points. BrdU was useful in assessing the short-term effects post-treatment. Six hours following treatment, it was evident that there was already an increased rate of DNA incorporation with the ACh and SP treated groups, and this was significantly reduced with co-administration of TGF-βRI/II kinase inhibitor. Crystal violet is a general DNA and protein stain and the results can be correlated with the number of cells present. Crystal violet was useful in testing for the medium-term effect of treatment as well as for indirect quantification of cell death. At the 24h time point, there was already significant increase for the TGF-β1 treated group relative to the control. On the other hand, the MTS assay, which is used to assess cell metabolic activity, was useful in testing for the long-term effects of treatment. While it did not show a significant difference between the groups at 24h, it did, however, show significant difference at 48h. The MTS assay was also useful in suggesting that the metabolism of the cells, which is often used as a surrogate of cell proliferation, continue to remain elevated following TGF-β1 treatment. TGF-β1 treatment on gingival fibroblasts has been shown to result in increased MTS as well as increased expression of the proliferating cell nuclear antigen (PCNA), supporting the suggestion that MTS is a surrogate of cell proliferation [21]. This may be important to support the notion that tenocytes are able to intrinsically regulate their own metabolism in an autocrine/paracrine fashion.

In our study, we did not study the role of ACh on nicotinic receptors. However, our previous study focusing on muscarinic receptors in tenocytes showed that cell proliferation were significantly diminished with atropine, the muscarinic acetylcholine receptor blocker [7]. It has also been shown that the type 2 muscarinic is the most prominent muscarinic acetylcholine receptor subtype to stimulate the proliferative rate of fibroblastic cells [22].

### Potential contribution of signal substances and TGF-β to tendon healing

During healing after acute tendon injuries, there is a marked proliferation and migration of local fibroblasts accompanied by increased expression of SP and TGF-β [23, 24]. Given that these substances regulate tenocyte proliferation as well as collagen synthesis, it has been hypothesized that SP and TGF-β are required for adequate tendon healing; however, this has not been clearly established, as it appears that tendon healing can occur even in the absence of SMAD signalling [25]. It could be hypothesised that SP and TGF-β are acute mediators involved in initial tendon healing; it is possible that sustained increased expression of SP and TGF-β could result in the characteristics seen in tendinosis such as hypercellularity and disorganized collagen. Therefore, it is important that the expression of SP and TGF-β is tightly regulated in the different phases of tendon healing.

### Proposed importance of TGF-β1 exerting negative feedback on mAChRs and NK-1R receptors

In our study, the exogenous administration of TGF-β1 resulted in decreased expression of all mAChRs as well as the NK-1R. This negative feedback mechanism could be presumed to be essential for normal tendon healing and function. In normal tendons, TGF-β1 expression is likely a tightly regulated process and dysregulation of it might contribute to the pathogenesis of tendinosis [14]. As discussed previously, SP and ACh have both been shown to cause increased TGF-β1 expression. In normal tendons, this process of activation is likely well controlled and TGF-β1 may exert negative feedback to decrease the effects of ACh and SP by decreasing the expression of mAChRs and NK-1R, respectively. This demonstrates the importance of maintaining a balance between the level of signal substances and their receptors. The importance of decreasing the expression of the mAChRs and NK-1R in response to high levels of TGF-β1 is likely beneficial to ensure that sustained tenocyte proliferation does not occur, which can be an undesirable effect tending towards tendinosis.

### The potential role of signal substances and TGF-β1 in the pathogenesis of tendinosis

It is previously shown that the expression of SP significantly increases following mechanical loading of tenocytes *in vitro* and *in vivo* [6, 8]. Based on the present study, we establish that SP results in an upregulation of TGF-β1 at the mRNA and protein level. Therefore, it is reasonable to speculate that the hypercellularity seen in tendinosis, as a result of increase in SP release, is mediated via TGF-β1. This line of reasoning would offer insight as to how both mechanical loading and TGF-β1 can both independently result in mediating cell proliferation and extracellular matrix synthesis in tenocytes as shown in a study by Mendias et al [26]. That is, mechanical loading results in a cascade of events that includes increased SP expression; SP in turn causes downstream upregulation of TGF-β1, which results in increased tenocyte proliferation.

Additionally, it could be postulated that in tendinosis, this feedback mechanism becomes aberrant, as SP [3–6], mAChR [2, 27], and NK-1R expression [3] may be elevated in tendinosis to drive the upregulation of TGF-β1 [28]. It is also demonstrated that intratendinous production of SP increases with mechanical loading and more importantly that this increase in SP precedes tendinosis [6]. Like SP, acute mechanical loading in human Achilles tendon can cause local elevation of TGF-β1 levels as shown in a microdialysis study [29]. Therefore, it could be extrapolated that SP would result in TGF-β1 release, which is the response that has been shown to occur in tendons during acute mechanical loading, as the active 25 kDa form of TGF-β1 is released from tendon and presumed to mediate a number of stress-induced tendon-growth and adaptation [12]. In tendinosis, it has been shown that TGF-β1 expression is significantly elevated in the parts of the tendon with major structural disorganisation as compared to the healthy parts of the tendon in patients with chronic Achilles tendinosis [30]. Elevated levels of TGF-β1 have been implicated in tendinosis [28] and based on our study, SP and ACh have the potential to mediate this process if the expression of these signal substances along with their preferred receptors are aberrant. Inhibiting TGF-β1 might be beneficial in decreasing the net accumulation of collagen in fibrotic tissue caused by sustained TGF-β1 expression, which results in dysregulation between deposition and degradation of extracellular matrix such as in carpal tunnel syndrome [31]. The results of the present study suggest that inhibiting SP and ACh will subsequently result in decreased expression of TGF-β1. This is likely beneficial as inhibition of TGF-β1 activity would promote a slower, albeit more organized, healing response [23].

## Conclusions

In summary, the present study is the first to demonstrate that the effects of ACh and SP on human tenocytes are mediated via TGF-β1. Our study shows (1) that ACh and SP induce upregulation of TGF-β1. Furthermore, we demonstrate that (2) TGF-β1 exerts proliferative properties in tenocytes and that (3) the proliferative effect of SP and ACh is significantly reduced in the presence of TGF-βRI/II kinase inhibitor. Lastly, (4) TGF-β1 downregulates the NK-1 R and mAChRs via a negative feedback mechanism. These findings suggest that the TGF-β1 axis is involved in the proliferative effects of ACh and SP on human tenocytes.
